# UV radiation increases phenolic compound protection but decreases reproduction in *Silene littorea*

**DOI:** 10.1371/journal.pone.0231611

**Published:** 2020-06-18

**Authors:** José Carlos Del Valle, Mª Luisa Buide, Justen B. Whittall, Fernando Valladares, Eduardo Narbona

**Affiliations:** 1 Departamento de Biología Molecular e Ingeniería Bioquímica, Universidad Pablo de Olavide, Seville, Spain; 2 Department of Biology, Santa Clara University, Santa Clara, California, United States of America; 3 Instituto de Recursos Naturales, Centro de Ciencias Medioambientales, CSIC, Madrid, Spain; United Arab Emirates University, UNITED ARAB EMIRATES

## Abstract

Plants respond to changes in ultraviolet (UV) radiation both morphologically and physiologically. Among the variety of plant UV-responses, the synthesis of UV-absorbing flavonoids constitutes an effective non-enzymatic mechanism to mitigate photoinhibitory and photooxidative damage caused by UV stress, either reducing the penetration of incident UV radiation or acting as quenchers of reactive oxygen species (ROS). In this study, we designed a UV-exclusion experiment to investigate the effects of UV radiation in *Silene littorea*. We spectrophotometrically quantified concentrations of both anthocyanins and UV-absorbing phenolic compounds in petals, calyces, leaves and stems. Furthermore, we analyzed the UV effect on the photosynthetic activity in hours of maximum solar radiation and we tested the impact of UV radiation on male and female reproductive performance. We found that anthocyanin concentrations showed a significant decrease of about 20% with UV-exclusion in petals and stems, and a 30% decrease in calyces. The concentrations of UV-absorbing compounds under UV-exclusion decreased by approximately 25% in calyces and stems, and 12% in leaves. Photochemical efficiency of plants grown under UV decreased at maximum light stress, reaching an inhibition of 58% of photosynthetic activity, but their ability to recover after light-stress was not affected. In addition, exposure to UV radiation did not affect ovule production or seed set per flower, but decreased pollen production and total seed production per plant by 31% and 69%, respectively. Our results demonstrate that UV exposure produced opposing effects on the accumulation of plant phenolic compounds and reproduction. UV radiation increased the concentration of phenolic compounds, suggesting a photoprotective role of plant phenolics against UV light, yet overall reproduction was compromised.

## Introduction

Ultraviolet (UV) radiation can both help and harm plants. Many flowering plants rely on UV nectar guides for pollination services [1]. Simultaneously, the high energy of UV radiation can be damaging to cells and presents a unique abiotic challenge to most land plants [2]. Furthermore, the “invisible” nature of UV radiation makes it particularly enigmatic at the ecological and physiological scales. UV-A (315–400 nm) and UV-B (280–315 nm) radiation has numerous positive and negative effects at the cellular and organismal scales [3–6], inducing a variety of morphological responses in plants [4,7]. In addition, UV-B radiation exerts damaging effects on DNA and chloroplasts, particularly photosystem II (PSII), and indirectly generates reactive oxygen species (ROS) that can further damage the photosynthetic apparatus [8,9].

Plants have developed a variety of mechanisms to avoid the harmful effects of UV radiation, mainly by filtering UV wavelengths, repairing UV-induced damage and quenching ROS [6,8,9]. Although the latter is primarily performed by antioxidant enzymes that control ROS levels [8,10], flavonoids and other phenolic compounds can detoxify ROS, as well [11–13]. Flavonoids are potent scavengers of ROS that prevent lipid peroxidation and scavenge free radicals, especially those flavonoids having a catechol group in the B-ring of the flavonoid skeleton (e.g. quercetin derivatives) [14,15]. Furthermore, exposure to excess light or UV-B radiation increases the synthesis of effective antioxidant dihydroxy B-ring-substituted flavonoids (e.g. luteolin derivatives) at the expense of other less effective antioxidant flavonoids (e.g. kaempferol derivatives) [16,17]. In addition to flavonoid’s key role as an antioxidant, other studies have implicated flavonoids in photoprotection by filtering the UV radiation [18,19]. For example, leaf epidermal flavonols play a predominant role in UV-B screening in *Secale cereale* and *Centella asiatica* [20,21].

Anthocyanins are flavonoid-based plant pigments that are synthesized in the last steps of the flavonoid biosynthetic pathway [22]. Anthocyanins mainly absorb in the green region of the visible (VIS) spectrum (500–565 nm), reducing the overall photosynthetically active radiation (PAR) (400–700nm) hitting the chloroplasts and facilitating rapid photosynthetic recovery following light stress [23–25]. In addition, when anthocyanins are acylated, they can absorb UV radiation, and are often the predominant phenolic compound responsible for ROS scavenging [26–29]. Moreover, UV stress is known to induce anthocyanin biosynthesis, which may contribute to the tolerance of UV radiation [30,31].

The aforementioned photoprotective functions of UV-induced flavonoids are not restricted to photosynthetic tissues, but also occur in floral structures such as anthers, ovaries, petals and sepals. Pollen grains accumulate flavonoids to protect them from UV-B damage and preserve their viability after anthesis [32], whereas flavonoids protect ovules by shielding ovaries from UV radiation [33]. In the same way, the accumulation of protective flavonoids in petals and sepals can reduce the damaging effects of UV radiation on these and other nearby reproductive tissues [34]. Additionally, petal flavonoids often form UV nectar guides for pollinators, thus UV-induced changes in petals could affect pollination success and thus, reproduction [1,35]. Furthermore, UV radiation may induce a variety of plant morphological responses in these reproductive structures. Many studies report a distinct flavonoid response following UV exposure dependent on the reproductive organ investigated (reviewed in [7]). For example, Koti et al. reported that UV-B radiation negatively affected a diversity of flower structures including flower size, pollen production, pollen germination and even pollen tube lengths in soybean (*Glycine max*) [36], and similarly decreased pollen and flower production in *Brassica rapa* [37].

Herein, we describe the effects of UV-radiation on the accumulation of plant phenolic compounds, gamete production and reproductive success of the shore campion (*Silene littorea* Brot., Caryophyllaceae). This annual species is endemic to coastal foredunes along the Iberian Peninsula and accumulates phenolic compounds (mostly flavones and anthocyanins derivatives) in petals, calyces, stems and leaves [38,39]. Our previous work has shown a latitudinal gradient in flavonoid accumulation that tends to increase from north to south in most plant tissues, correlated with increased solar exposure and temperature [39]. Moreover, we found that intense solar radiation, including UV and VIS spectra, increased the synthesis of flavones and anthocyanins in most aboveground tissues of *S*. *littorea* [40]. In the current study, we focus on the effect of the UV irradiation on flavonoid accumulation in this species. We spectrophotometrically quantified the concentrations of anthocyanins and UV-absorbing compounds in petals, calyces, leaves and stems of plants grown with or without exposure to UV radiation. Then, we analyzed the effects on photosynthetic efficiency and compared it to male and female reproductive output.

Flavonoids have a key role in photoprotection [15,19], but the synthesis of these phenolic compounds may represent a cost for the plant [24]. Consequently, we predict that the exclusion of UV radiation will result in a decrease in UV-inducible flavonoid concentrations in all tissues. This energetic and carbon savings under UV-exclusion may result in increased reproductive allocation [41]. In contrast, without UV protection, we predict that photodamage will decrease photosynthetic activity [9,42] resulting in lower reproductive output. Since *S*. *littorea* inhabits exposed coastal dunes habitats with high solar radiation levels, we hypothesize that this species will have an effective light-stress recovery system that prevents long-term photoinhibition.

## Materials and methods

### Study system and experimental design

**Fig 1:**
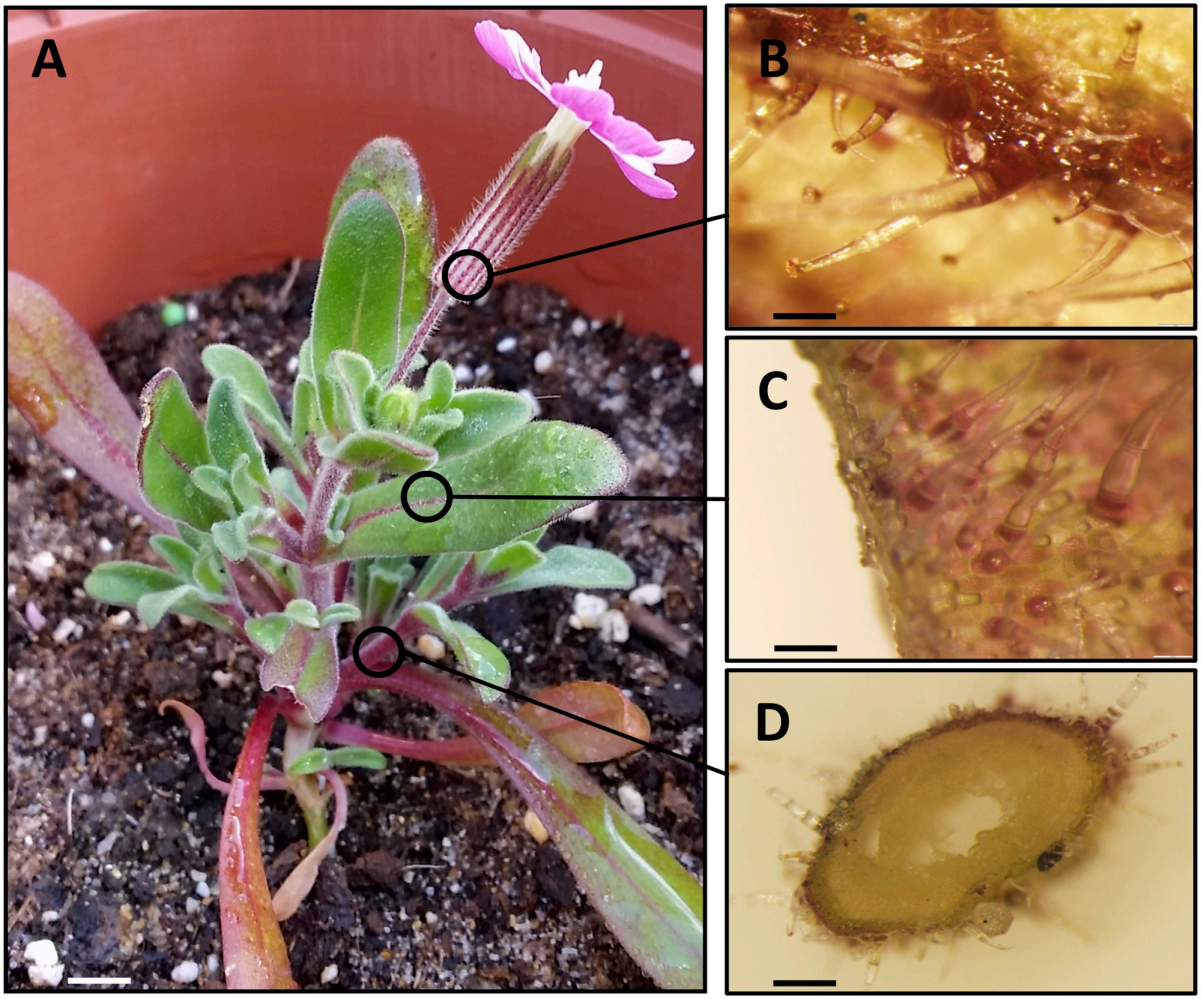
Detailed view of a *Silene littorea* plant (A) showing the accumulation of anthocyanins throughout the whole plant. Stereo-microscope photographs show pigments in the surface of the calyx ribs (B), adaxial surface of the leaf (C), and cross section of a stem (D). Scale bars: 5 mm (A), 0.5 mm (B, C), and 1 mm (D).

*Silene littorea* is an annual plant that accumulates anthocyanins (cyanidin derivatives) and flavones (mainly isovitexin and isoorientin derivatives) in both reproductive and vegetative tissues [38] (Fig 1). This species inhabits coastal populations from the northwestern corner to the southeastern portion of the Iberian Peninsula [39]. We collected seeds from six plants from a northwestern population (Furnas; 42° 38' 15'' N, 9° 2' 21'' W) and six plants from a southwestern population (Sines; 37° 55' 17'' N, 8° 48' 17'' W). No permissions were required to sample at both field locations (*S*. *littorea* is an abundant plant at these locations and is not a protected species). The degree of solar irradiance is 30% higher in the southern population because it is approximately 500 km closer to the equator when compared to the northern population [39].

Seeds obtained from the 12 maternal families were scarified and maintained at 45 °C for a month to break dormancy, and afterwards they were transferred to a germination chamber set to 22 °C/15 °C (12 h light/12 h dark). The resulting seedlings were planted in pots filled with 2.5 L of a mixture of standard substrate (80–90% organic material, pH = 6.5) and beach sand (v:v 50:50) and were grown in the greenhouse at Pablo de Olavide University (Seville, Spain) for one month (climatic conditions of the experimental area are shown in S1 Fig). Given the low germination rates of this species and the high mortality at the seedling stage, the final number of surviving seedlings was 65 (belonging to nine maternal families; five from Furnas and four from Sines). On February 8, 2016 (approximately one month before flowering), pots were put outside on two benches in an experimental garden until June 20, 2016. This period covers the entire flowering period. Plants that shared the same maternal family were equally assigned to either treatment (bench) whenever possible. A total of 41 and 24 plants were assigned to the UV-present and UV-exclusion treatments, respectively (see S1 Table for more details about the distribution of plants from the same maternal family in the two UV-treatments). The position of the plants was randomly changed every two weeks to minimize microenvironmental effects. The bench assigned to the UV-present treatment was covered with a methacrylate filter that transmitted a significant portion of the UV irradiance, especially wavelengths greater than 335nm (transmittance of 20% and 95% of the UV-B and UV-A radiation, respectively), whereas no wavelengths were blocked above the UV range (>400nm). The bench assigned to the UV-exclusion treatment was covered with a polycarbonate filter preventing most UV radiation below the 385nm (transmittance of 7% and 25% of the UV-B and UV-A radiation, respectively); the polycarbonate filter did not absorb light above 385nm (S2 Fig). Total transmittance of the two treatments was reduced by 9.2% in the UV-present and 22.3% in the UV-exclusion treatment when compared to natural sunlight at this location. Maximum solar irradiation of natural sunlight was 1258 W/m^2^ and UV-A/B irradiance was 43.6 W/m^2^. Measurements were taken at 2 PM on a sunny day (June 6, 2016). Total solar radiance and UV were measured by means of Megger PVM210 irradiance meter (range sensitivity = 1999 W/m^2^; resolution = 0.1 W/m^2^) (Megger Co., Dallas, USA) and PCE-UV34 UV light meter (range sensitivity = 0.000 to 199.9 W/m^2^; resolution = 0.01 W/m^2^) (PCE Inst., Durham, UK), respectively. Total solar UV irradiance during maximum solar radiation (∼2:30 PM) increased about 86.1% during the experiment, ranging from 25.8 ± 1.79 in February to 48.0 ± 0.84 Wh/m^2^ in June (mean ± SE; S2 Table). Likewise, plants’ exposure to natural sunlight increased from approximately seven to 11 hours during the experimental time period (S1 Fig).

### Quantification of phenolic compounds

During peak flowering (May 2016), we collected five petals and the calyx of the same flower, mid-stem leaf and a 1 cm stem section from the main axis from 34 and 22 plants grown in the UV-present and UV-exclusion environments, respectively (S1 Table). Samples were extracted in 1.5 ml of methanol containing 1% of HCl following the procedure described in Del Valle et al. [39]. Three replicates of 200 μL per sample extraction were used to estimate concentrations of plant phenolic compounds on a Multiskan GO microplate spectrophotometer (Thermo Fisher Scientific Inc., MA, USA). In particular, anthocyanins and UV-absorbing compounds were quantified at A_520_ and A_350_, respectively. In photosynthetic organs (calyces, leaves and stems), anthocyanin concentration was corrected as A_520_ - (0.24 x A_653_) to compensate for the small overlapping absorption by chlorophyll [43]. Since flavones (isovitexin and isoorientin derivatives) constitute the predominant UV-absorbing compounds detected in methanol extracts of floral and photosynthetic tissues of *S*. *littorea* [38], total phenolic compounds detected at A_350_ were inferred to be primarily flavones. Anthocyanin and UV-absorbing compound concentrations were calculated following Del Valle et al. [38] and expressed as milligrams of cyanidin-3-glucoside, isovitexin and isoorientin equivalents per gram fresh weight, respectively.

### Assessment of photosynthetic activity

To determine if there were physiological consequences of plants grown with and without UV radiation, the photochemical efficiency of PSII (*Fv/Fm*) was measured in calyces and leaves of 30 plants from Sines (14 and 16 from the UV-present and UV-exclusion treatments, respectively) using a portable pulse-modulated chlorophyll fluorometer (FMS2, Hansatech Instruments, Norfolk, UK). Measurements were carried out before dawn (∼7 AM) and during maximum solar radiation (∼2:30 PM) at two dates during the experiment—during early flowering (March) and at peak flowering (May). To assess the physiological status of photosynthetic tissues across the experiment, measurements were carried out on fully exposed plants over two sunny days [44]. To minimize temporal variation in *Fv/Fm*, all measurements were made within one hour of each other. Prior to taking physiological measurements, samples were acclimated for 30 minutes in the dark using leaf-clips that contained a mobile shutter.

### Assessment of plant reproductive performance

Flower and fruit production in 41 and 24 plants from the UV-present and UV-exclusion treatments were monitored weekly during the entire flowering period, from March 10 to June 20, 2016. Individual flowers were surveyed for either fruit production or fruit abortion to determine the proportion of flowers that set fruit. In May, each plant with at least one successful fruit had a single capsule collected. A total of 33 and 21 mature fruits were collected from plants growing in the UV-present and UV-exclusion treatments, respectively. For each mature fruit, their seeds and aborted ovules were counted under the dissecting microscope to calculate the proportion of ovules that set seed. Then, we estimated seed production per plant for all the plants from which we collected a mature fruit as the product of seeds/fruit x total number of fruits produced. Pollen generation and ovule production were analyzed following the procedure described in Narbona et al. [45] from unopened flower buds preserved in FAA (95% ethanol, dH_2_O, 37–40% formaldehyde, glacial acetic acid, 10:7:2:1) from nine and 13 plants grown in the UV-present and UV-exclusion treatments, respectively. The total number of pollen grains per anther was calculated as the average number of pollen grains counted in one upper and one lower anther of an unopened flower bud per plant.

### Statistical analysis

Generalized linear mixed models (GLMMs) with Gaussian link functions were used to test the effect of UV radiation on the accumulation of phenolic compounds (anthocyanins and UV-absorbing compounds) in each plant tissue. We considered treatment and source population as fixed factors and maternal family as a random factor. Plant phenolic concentrations were log-transformed prior to conducting the GLMMs analyses using the R-package “lme4” [46]. We performed Tukey Post-Hoc analyses to make pairwise comparisons between UV-present and UV-exclusion treatments, and then the “cld” (compact letter display) function was used to show differences between populations. Post-Hoc analyses were carried out using the “multcomp” R-package [47]. Due to the low number of experimental plants, we used the conservative Bonferroni adjustment of p-values in pairwise comparisons [48]. The same analyses were used to test for differences in male and female reproductive performance and in the photochemical efficiency of PSII (*Fv/Fm*) between plants grown in the different UV treatments. For the latter analysis, independent comparisons were done for leaves and calyces and in the early flowering (March) and peak flowering (May) periods, as well as pairwise comparisons of the photochemical efficiency between predawn and afternoon conditions. Pearson’s correlations with a Bonferroni adjustment for multiple comparisons were used to assess the relationship between the production of phenolic compounds and male and female reproductive output [49]. All analyses were performed in R v3.4.0 [50].

## Results

### Effects of UV radiation on phenolic compound production

**Fig 2:**
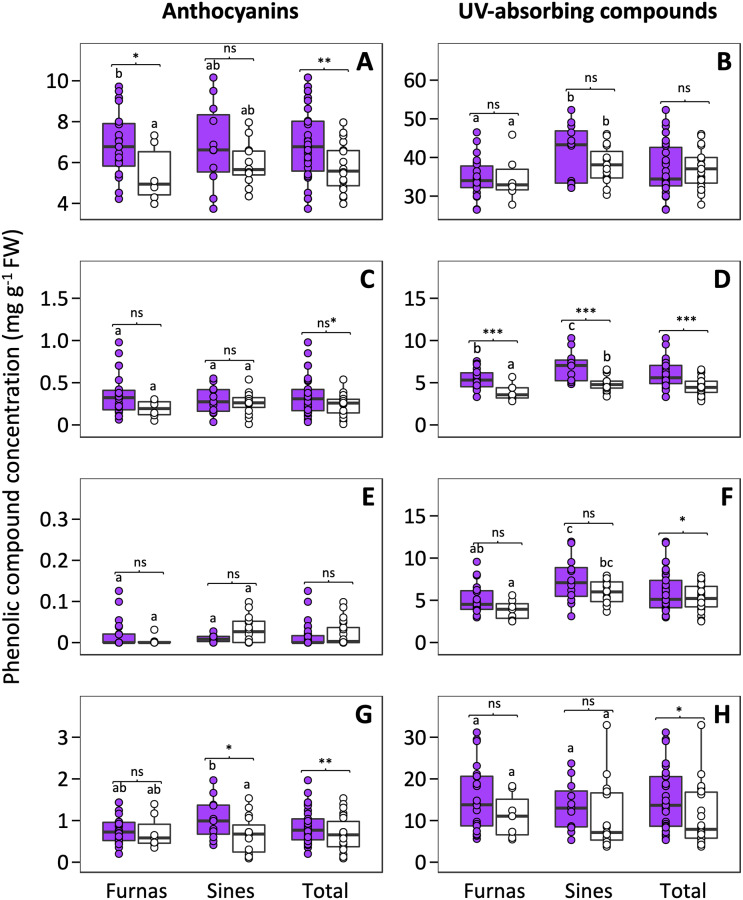
Boxplots representing anthocyanin and UV-absorbing compound concentrations in the UV-present (purple boxes) and UV-exclusion (white boxes) treatments in petals (A, B), calyces (C, D), leaves (E, F) and stems (G, H). The central line displays the median, the bottom and top of the box are the first and third quartiles, and dots represent sample values. Lowercase letters are used to show statistical results of multiple comparisons between populations. Within each population, pairwise comparisons between light treatments using Bonferroni adjustment are shown. FW, fresh weight; ns, not significant; ns*, marginally significant; *, *P* < 0.05; **, *P* < 0.01; ***, *P* < 0.001.

In general, plants from the UV-exclusion treatment showed lower concentrations of anthocyanins, but this decrease was not consistent across all tissues. Specifically, anthocyanin concentrations in petals and stems statistically decreased about 20% in these plants, whereas in calyces there was a 30% decrease and the differences were only marginally significant (Fig 2, Table 1). Anthocyanins were nearly absent altogether in leaves (Fig 2E). In plants from the UV-exclusion treatment, the concentration of UV-absorbing compounds decreased in leaves (12%), calyces (23%), and stems (25%) when compared to the UV-present treatment, but in petals the difference was not significant (Table 1).

**Table 1:** Results from GLMMs testing the effect of UV radiation, population and their interaction on the production of anthocyanins and UV-absorbing compounds in each plant tissue.

		Anthocyanins				UV-absorbing compounds			
Tissue	Source of variation	SS	Denominator d.f.	*F*	*P*	SS	Denominator d.f.	*F*	*P*
**Petals**	Treatment	0.670	46.62	7.968	**0.007**	0.019	38.78	1.469	0.233
	Population	0.033	19.79	0.396	0.536	0.477	42.86	36.15	**<0.001**
	Treatm. x Pop.	0.096	47.86	1.140	0.291	0.001	38.68	0.004	0.949
**Calyces**	Treatment	0.674	44.48	3.729	0.059	1.013	44.39	37.27	**<0.001**
	Population	0.121	43.22	0.667	0.419	0.606	46.06	22.29	**<0.001**
	Treatm. x Pop.	0.231	45.11	1.276	0.265	0.018	44.94	0.647	0.426
**Leaves**	Treatment	0.007	46.70	0.176	0.677	0.368	49.00	5.145	**0.028**
	Population	0.078	23.18	1.899	0.181	1.166	49.00	16.28	**<0.001**
	Treatm. x Pop.	0.199	48.29	4.840	**0.033**	0.023	49.00	0.327	0.570
**Stems**	Treatment	1.572	44.65	8.527	**0.005**	1.295	46.10	6.203	**0.016**
	Population	0.035	49.27	0.191	0.664	0.049	42.77	0.237	0.629
	Treatm. x Pop.	0.339	45.22	1.837	0.182	0.008	46.92	0.041	0.841

Significant *P*-values were highlighted in bold.

Numerator d.f. = 1 in all cases.

Sines and Furnas populations did not show significant differences in anthocyanin concentrations in any of the sampled tissues (Table 1). Conversely, the UV-absorbing compound concentrations were significantly higher in plants from Sines in all tissues except for the stems (Fig 2, Table 1), and the interactions of light treatment and population were not significant (i.e. the decrease of UV-absorbing compound concentration in plants experiencing UV-exclusion was similar in both populations).

When we analyzed each population independently, we found that the only significant differences in anthocyanin concentrations between treatments were in petals of plants from Furnas and in stems of plants from Sines (Fig 2A and 2G). With respect to UV-absorbing compounds, the only significant differences between treatments were found in calyces of plants from both populations (Fig 2D). Interestingly, plants from both UV-exclusion and UV-present treatments of Sines showed higher levels of UV-absorbing compounds than their respective treatments in Furnas.

### Effects of UV radiation on photosynthetic performance

**Fig 3:**
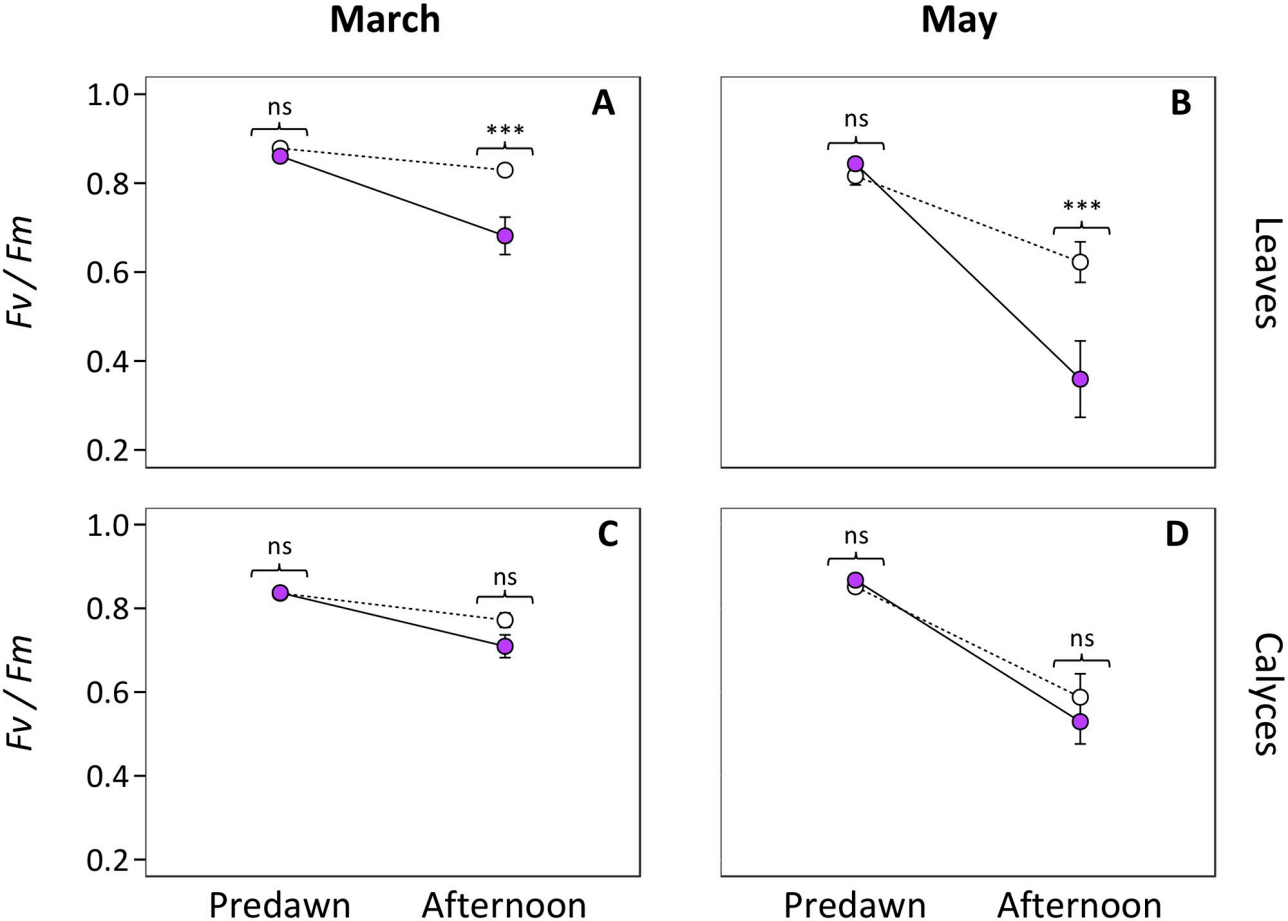
Variation of photochemical efficiency (*Fv*/*Fm*) from predawn conditions to afternoon. The mean *Fv/Fm* values from leaves (A, B) and calyces (C, D) in the early flowering (March; A and C) and peak flowering (May; B and D) periods are shown. Plants from the UV-present treatment are displayed by pink filled circles and solid lines, whereas those from the UV-exclusion treatment are displayed with empty circles and dashed lines. Statistical results of independent pairwise comparisons using Bonferroni adjustment between UV treatments in predawn and afternoon conditions are displayed. ns, not significant; ***, *P* < 0.001. Error bars represent ± SE.

Plants decreased their photochemical efficiency (*Fv/Fm*) from 6% to 58% in the afternoon following maximum exposure to light stress. However, in the predawn estimates, after an entire night of recovery, they showed similar *Fv/Fm* values ranging from 0.82 to 0.88 (Fig 3, Table 2). Leaves showed significant differences in their photochemical efficiency between UV-treatments and between measurement conditions (predawn or afternoon), and the interaction of UV-treatments and measurement conditions was also significant (Table 2). In the afternoon, leaves of the UV-present treatment showed a 20.8% and 57.4% reduction of *Fv/Fm* values in early flowering (March) and peak flowering (May) timepoints, respectively (*P* < 0.001 for both pairwise comparisons, Table 3; Fig 3A and 3B). The observed decrease in afternoon leaf *Fv/Fm* values of the UV-present treatment paralleled a 10.4% increase in UV irradiance during hours of maximum solar radiation (43.8 and 48.4 Wh/m^2^ in March and May measurements of the photochemical efficiency of PSII, respectively; S3 Fig). In calyces, statistical differences in their photochemical efficiency were found only between measurement conditions (predawn or afternoon) in March and May (Table 2). Pairwise comparisons in calyces revealed significant lower *Fv/Fm* values in afternoon conditions, regardless of the UV treatment or the flowering period (*P* < 0.032, Table 3; Fig 3C and 3D).

**Table 2:** Results from GLMMs testing the effect of UV radiation, measurement condition (predawn or afternoon) and their interaction on the photochemical efficiency of PSII (*Fv/Fm*) in leaves and calyces.

Tissue	Stage	Source of variation	SS	Denominator d.f.	*F*	*P*
Leaves	Early flowering (March)	Treatment	0.030	50.00	19.07	**< 0.001**
		Measurement condition	0.056	50.00	34.86	**< 0.001**
		Treatm. x Measurement condition	0.020	50.00	12.34	**< 0.001**
	Peak flowering (May)	Treatment	0.083	40.00	6.209	**0.017**
		Measurement condition	0.528	40.00	39.50	**< 0.001**
		Treatm. x Measurement condition	0.115	40.00	8.614	**0.006**
Calyces	Early flowering (March)	Treatment	0.004	32.39	2.822	0.103
		Measurement condition	0.039	47.25	30.42	**< 0.001**
		Treatm. x Measurement condition	0.004	47.25	3.401	0.071
	Peak flowering (May)	Treatment	0.001	40.39	0.182	0.672
		Measurement condition	0.375	38.80	48.32	**< 0.001**
		Treatm. x Measurement condition	0.005	38.80	0.639	0.429

Significant *P*-values were highlighted in bold.

Numerator d.f. = 1 in all cases.

**Table 3:** Comparisons of the photochemical efficiency (*Fv/Fm*) between predawn and afternoon conditions. Pairwise comparisons were independently performed in leaves and calyces from the UV-exclusion and UV-present treatments and either in the early flowering (March) and peak flowering (May).

Tissue	Stage	Treatment	Estimate	Std. Error	*Z* value	*P*
Leaves	Early flowering (March)	UV-exclusion	-0.027	0.014	-1.873	0.239
		UV-present	-0.104	0.017	-6.117	**< 0.001**
	Peak flowering (May)	UV-exclusion	-0.117	0.047	-2.480	0.063
		UV-present	-0.323	0.052	-6.252	**< 0.001**
Calyces	Early flowering (March)	UV-exclusion	-0.037	0.013	-2.788	**0.032**
		UV-present	-0.073	0.015	-4.889	**< 0.001**
	Peak flowering (May)	UV-exclusion	-0.163	0.035	-4.611	**< 0.001**
		UV-present	-0.205	0.039	-5.202	**< 0.001**

Significant *P*-values were highlighted in bold.

### Effects of UV radiation in reproductive performance

**Fig 4:**
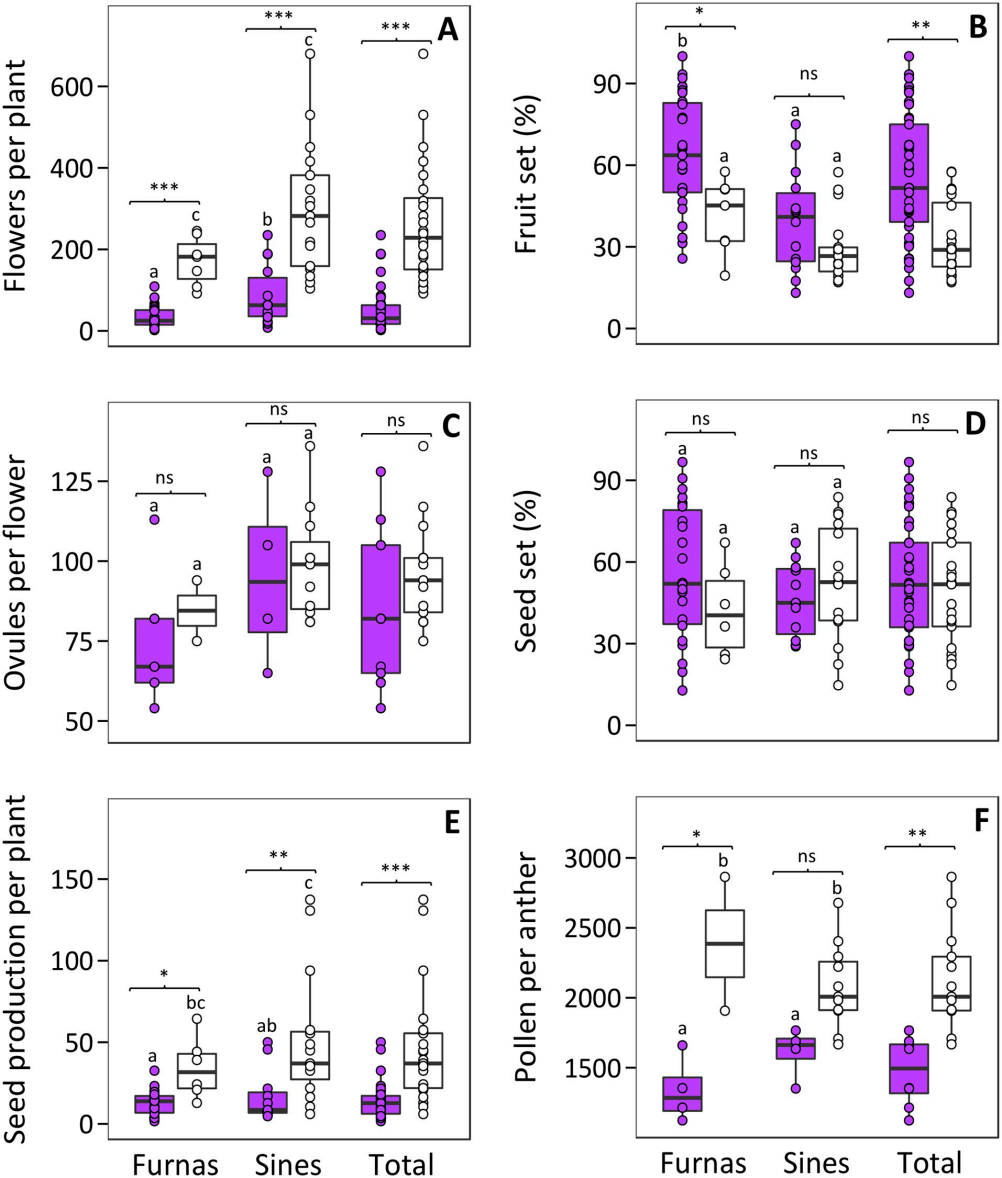
Boxplots representing the total flowers per plant (A), fruit set (B), ovules per flower (C), seed set (D), seed production per plant (E) and pollen per anther (F) in plants growing in the UV-present (purple boxes) and UV-exclusion (white boxes) treatments. Points represent values for all estimations of plant reproductive performance. The central line displays the median, the bottom and top of the box are the first and third quartiles, and point represent sample values. Lowercase letters are used to show statistical results of multiple comparisons between populations. Within each population, pairwise comparisons between light treatments using Bonferroni adjustment are shown. ns, not significant; *, *P* < 0.05; **, *P* < 0.01; ***, *P* < 0.001.

There was a significant difference in flower production between the two experimental conditions (Table 4). Plants from the UV-exclusion treatment displayed approximately five times more flowers than those with UV-present (261.4 ± 30.1 and 50.7 ± 8.3, respectively; mean ± SE; Fig 4A). In addition, flower production was significantly different between the two populations, being approximately two times higher in Sines plants. Conversely, fruit set was nearly double in the UV-present treatment and 57% higher in plants from Furnas population (Fig 4B, Table 4). The number of ovules per flower and seed set were not significantly different between light treatments, nor between source populations (Fig 4C and 4D). The total seed production per plant was approximately three times higher in plants from the UV-exclusion treatment compared to the UV-present plants (46.2 ± 7.8 and 14.3 ± 1.9, respectively; Fig 4E), and did not show statistical differences between populations (Table 4). Pollen production decreased by ~31% in plants exposed to UV radiation (2126.1 ± 99.0 and 1473.9 ± 85.8, respectively; Fig 4F), but no significant difference between the two populations was detected. The interactions of UV treatment and population were not significant for any of the studied reproductive outputs (Table 4).

**Table 4:** Results from GLMMs testing the effect of UV radiation, population and their interaction on the estimations of male and female reproductive performance in *S*. *littorea*.

	Source of variation	SS	Denominator d.f.	*F*	*P*
Flowers per plant	Treatment	37.55	52.45	58.04	**< 0.001**
	Population	5.040	6.604	7.789	**0.029**
	Treatm. x Pop.	0.648	60.01	1.002	0.321
Fruit set	Treatment	1.457	61.00	9.476	**0.003**
	Population	2.405	61.00	15.64	**< 0.001**
	Treatm. x Pop.	0.112	61.00	0.725	0.398
Ovules per flower	Treatment	0.020	12.21	0.524	0.483
	Population	0.050	17.02	1.282	0.273
	Treatm. x Pop.	0.007	12.55	0.183	0.677
Seed set	Treatment	0.056	48.29	0.267	0.608
	Population	0.001	27.29	0.009	0.924
	Treatm. x Pop.	0.140	49.75	0.666	0.418
Seeds production per plant	Treatment	11.42	50.00	21.48	**< 0.001**
	Population	0.401	50.00	0.755	0.389
	Treatm. x Pop.	0.012	50.00	0.022	0.883
Pollen per anther	Treatment	0.616	17.00	25.32	**< 0.001**
	Population	0.004	17.00	0.158	0.696
	Treatm. x Pop.	0.093	17.00	3.807	0.067

Significant *P*-values were highlighted in bold.

Numerator d.f. = 1 in all cases.

When assessing the relationship between plant phenolic compound production and male and female reproductive outputs, we did not find any significant correlations after applying a Bonferroni-correction (S3 Table).

## Discussion

### Effects of UV radiation on phenolic compound production

We found that exposure to UV radiation led to a generalized increase in the concentration of anthocyanin and UV-absorbing compounds in *S*. *littorea*, suggesting that part of this plant’s response to UV stress is an increase in total phenolic compound concentration. In a previous study, HPLC-DAD-MS^n^ analysis of the main phenolic compounds in aboveground tissues of *S*. *littorea* showed that all anthocyanins are cyanidin derivatives, whereas the most abundant UV-absorbing compounds are two flavones: *C*-glycosides of apigenin (isovitexin derivatives) in petals and *C*-glycosides of luteolin (isoorientin derivatives) in photosynthetic tissues [38]. In this study, we inferred that the main phenolic compounds detected spectrophotometrically are the same than those described previously by Del Valle et al. [38] for this species. Isoorientins are dihydroxy B-ring-substituted flavonoids, which are known to have efficient antioxidant properties [14,51–53]. This UV-induced accumulation of flavonoids is a common antioxidant response in plants [4,54]. In addition, most flavones in *S*. *littorea* (i.e. isovitexin and isoorientin derivatives) are predominantly linked to hydroxycinnamic acids such as ferulic, caffeic or *p*-coumaric acids [38], which are known to enhance flavonoid absorption in the UV-A and UV-B wavelengths [28,55–57]. Similarly, the leaves of purple basil (*Ocimum basilicum*) accumulate coumaroyl anthocyanins that are more responsive to quenching sunlight irradiance, mainly in the UV-B wavelength, than non-acylated anthocyanins [27]. Although we did not obtain direct evidence of the biochemical constitution of these samples, based on previous studies in *S*. *littorea*, we infer that this species exhibits a robust biochemical toolkit that may protect itself from the oxidative stress caused by UV radiation in open, exposed beach dune environments.

Despite differences in phenolic compound production caused by UV radiation, plants protected from UV radiation still accumulated measurable amounts of anthocyanins and UV-absorbing compounds (likely flavones) in most aboveground tissues. The accumulation of these phenolic compounds may have been induced by high levels of PAR on these plants. In *Brassica oleracea*, for example, the concentration of UV-absorbing quercetin increases in line with PAR levels [58]. Similarly, high levels of PAR might cause an initial response of synthesis of anthocyanins and flavones in *S*. *littorea*, whose concentrations could be increased if plants are subsequently exposed to UV radiation. However, given that anthocyanins and flavones perform a plethora of protective functions against many biotic and abiotic factors [28,59,60], they could be performing non-photoprotective functions. For example, petal isovitexins of *Silene latifolia* help regulate vacuole homeostasis in epidermal cells, preventing petals from wilting [61]. In addition, flavones are produced constitutively in aboveground tissues of *S*. *littorea* when plants grow in low light levels conditions [40]. Thus, we cannot rule out that the selective pressures of other biotic and abiotic agents could explain the constitutive accumulation of phenolic compounds found in *S*. *littorea* plants protected from UV radiation.

The increased concentration of phenolic compounds in response to UV radiation was not homogeneous across tissues: petals respond to UV by increasing anthocyanins, calyces and leaves respond by increasing UV-absorbing compounds, and stems through both anthocyanin and UV-absorbing compounds. This result is not surprising because the regulation of flavonoid biosynthesis is tissue-specific [62]. The depletion of anthocyanins in petals in the UV-exclusion treatment translates into a change in color intensity [63], which may be differentially perceived by insect pollinators [64]. On the other hand, calyces of plants from both UV-exclusion and UV-present treatments of the Sines population showed higher levels of UV-absorbing compounds than those of Furnas in each treatment. This difference may reflect local adaptation of the Sines population to the higher UV radiation compared with the Furnas population [39]. However, further studies are necessary to assess whether flavonoid biosynthesis in *S*. *littorea* shows signals of local adaptation in the wild across the UV radiation gradient across its distribution area.

### Effects of UV radiation on photosynthetic performance

*Silene littorea* showed a more substantial decline of the quantum efficiency of PSII when plants were exposed to UV stress, especially in leaves. Previous studies have shown that sunlight’s UV region is essential for photoinhibition of PSII of leaves. For example, Albert et al. [65] demonstrated that PSII performance and net photosynthesis in *Salix arctica*, is negatively affected by the ambient solar UV-B radiation. Given that *S*. *littorea* was more susceptible to photoinhibition when it was exposed to UV stress, our findings suggest that ambient solar UV radiation is a significant source of stress on the photosynthetic activity of these plants.

Despite the negative effects of UV stress on the photosynthetic activity in *S*. *littorea*, this species seems to have an optimal light-stress recovery system. This species does not incur chronic photoinhibition, since after relaxation of photoinactivation its *Fv/Fm* values were within the range for healthy plants (0.74–0.85) [66]. The photoprotection mechanism of plants involves a variety of defenses against light-induced ROS, including the synthesis of antioxidant anthocyanins and flavonoids [12,13]. In this regard, dihydroxy B-ring-substituted flavonoids located in the chloroplasts help antioxidant enzymes to reduce light-induced ROS and ROS diffusing out of the chloroplast are scavenged by vacuolar flavonoids [15]. In addition, leaves accumulating anthocyanins incur less photoinhibition after a saturating light stress as compared with green leaves [27,67]. We hypothesized that phenolic compounds of *S*. *littorea* may contribute to photoprotection necessary to thrive in habitats with highly solar radiation such as coastal foredunes along the Iberian Peninsula [39]. In the same way, anthocyanin accumulation depends on sunlight in *Silene germana*, helping to prevent the oxidative stress caused by the excessive summer sunlight [68].

### Effects of UV radiation on reproductive output

Plants exposed to UV radiation produced approximately three times less total number of seeds per plant than those shielded from UV, driven primarily by a decrease in total flower production. In a previous study, we found that flower production in *S*. *littorea* increases as a consequence of high natural sunlight levels [69], but exposure to sunlight also entails the exposure to harmful UV wavelengths. Here, we demonstrated that the absence of these harmful effects in the UV-exclusion treatment facilitates the absorption of PAR and enhances flower production. Although many studies often report enhanced flowering when plants are exposed to supplemental UV radiation (e.g. [70,71]), other studies have reported the opposite effect (e.g. [70,71]). Additionally, we found that the proportion of flowers yielding fruits was nearly double in plants under UV stress. Even though other studies have reported increasing fecundity in plants exposed to moderate UV radiation [72], we suggest that significant differences in fruit set between light treatments could be influenced by the resources allocated to the high flower production of plants growing in the absence of UV stress.

The decrease in pollen production detected in plants exposed to UV light is consistent with results from other species [36,37,73]. Conversely, ovule production was similar in plants from both light treatments. Ovules occur in ovaries, which are well protected against UV stress due to their accumulation of UV-absorbing compounds that attenuate UV radiation [7,33]. In *S*. *littorea*, upper anthers occur slightly beyond the corolla opening at anthesis and therefore have higher exposure to UV radiation, whereas carpophore is embedded in the calyx. Thus, ovule production is less likely to be compromised by solar radiation since ovules are protected from UV radiation by several layers of tissue.

## Conclusions

We propose that the production of phenolic compounds (both anthocyanins and UV-absorbing compounds) was activated as a defense mechanism against UV radiation, which may prevent chronic photoinhibition and promote rapid photosynthetic recovery. Conversely, exposure to UV radiation significantly decreased flower and pollen production in this species. There may be a balance between protection and reproduction especially important in the exposed coastal foredune habitat. Thus, the allocation of metabolic resources may provide an efficient photoprotective toolkit and, at the same time, guarantee reproduction in this species in Mediterranean climates subjected to high levels of UV radiation.

## Supporting information

S1 Table Number of plants for each maternal genotype, population and treatment (UV-present and UV-exclusion treatments). The number of plants sampled for anthocyanin and UV-absorbing compound concentration is indicated in parentheses.
(DOCX)

S2 Table Average UV-A/B radiation doses for the experimental area in hours of maximum solar irradiance (∼2:30 PM) from February to June 2016. Data from HelioClim-3 database was provided by SoDa service.
(DOCX)

S3 Table Pearson correlation coefficients for the comparison between plant phenolic compound production (anthocyanins and UV-absorbing compounds) in each plant tissue and reproductive outputs of *S*. *littorea*. (DOCX)

S1 Fig Historical climatic conditions (1981–2010) from February to June at the Seville airport (37° 25' 0'' N, 5° 52' 45'' W), separated 8 km from the experimental area. Filled circles, average temperature (°C); filled squares, maximum temperature (°C); empty circles and dashed line, relative humidity (%); empty squares and dashed line, precipitation (mm); bars, daytime duration (hours/day). Data from AEMET (State Meteorological Agency from Spain) database.
(TIFF)

S2 Fig Comparisons of light reaching plants under each UV treatment. Methacrylate filter used in the UV-present treatment (purple line) allowed for the transmittance of significant portion of the UV irradiance, especially over the 335nm. Polycarbonate filter used in the UV-present treatment (green line) blocked the transmittance of UV wavelengths until the 385 nm, approximately. Natural sunlight is represented with a black line. Transmittance were measured using the portable spectrophotometer described in [63].
(TIFF)

S3 Fig UV radiation (280-400nm) reaching plants in the experimental garden along the experiment. Blue and red lines represent UV radiation in predawn (7 AM) and in the afternoon (3 PM), respectively; triangles represent days when we performed the measurements of the photochemical efficiency of PSII (*Fv/Fm*) in the early flowering (March) and peak flowering (May). Data from HelioClim-3 database was provided by SoDa service.
(TIFF)
