# Supplementary material for: UV radiation increases phenolic compound protection but decreases reproduction in Silene littorea
Source: PLoS One. 2020 Jun 18;15(6):e0231611. doi: 10.1371/journal.pone.0231611 (PMC7302690; doi:10.1371/journal.pone.0231611)
Supplement: S1 Table — The number of plants sampled for anthocyanin and UV-absorbing compound concentration is indicated in parentheses. (DOCX) [file pone.0231611.s001.docx]

**Supporting information**

| **S1 Table.** **Number of plants for each maternal genotype, population and treatment (UV-present and UV-exclusion treatments).** The number of plants sampled for anthocyanin and UV-absorbing compound concentration is indicated in parentheses. | | | |
| --- | --- | --- | --- |
| **Population** | **Maternal family** | **Treatment** | |
|  |  | **UV-present** | **UV-exclusion** |
| Sines | 1 | 4 (3) | 3 (3) |
|  | 2 | 5 (4) | 4 (4) |
|  | 3 | 0 (0) | 1 (1) |
|  | 4 | 5 (5) | 9 (7) |
| Furnas | 5 | 2 (1) | 2 (2) |
|  | 6 | 2 (2) | 0 (0) |
|  | 7 | 6 (5) | 2 (2) |
|  | 8 | 4 (4) | 0 (0) |
|  | 9 | 13 (10) | 3 (3) |
| Total | 9 | 41 (34) | 24 (22) |
