# Supplementary material for: UV radiation increases phenolic compound protection but decreases reproduction in Silene littorea
Source: PLoS One. 2020 Jun 18;15(6):e0231611. doi: 10.1371/journal.pone.0231611 (PMC7302690; doi:10.1371/journal.pone.0231611)
Supplement: S2 Table — Data from HelioClim-3 database was provided by SoDa service. (DOCX) [file pone.0231611.s002.docx]

**Supporting information**

| **S2 Table. Average UV-A/B radiation doses for the experimental area in hours of maximum solar irradiance (∼2:30 PM) from February to June 2016.** Data from HelioClim-3 database was provided by SoDa service. | |
| --- | --- |
| **Month** | **Average ± S.E.** |
| February (8-29) | 25.8 ± 1.70 |
| March | 37.2 ± 1.01 |
| April | 35.3 ± 1.90 |
| May | 43.2 ± 1.59 |
| June (1-20) | 48.0 ± 0.84 |
