# Supplementary material for: UV radiation increases phenolic compound protection but decreases reproduction in Silene littorea
Source: PLoS One. 2020 Jun 18;15(6):e0231611. doi: 10.1371/journal.pone.0231611 (PMC7302690; doi:10.1371/journal.pone.0231611)
Supplement: S3 Table — (DOCX) [file pone.0231611.s003.docx]

**Supporting information**

| **S3 Table. Pearson correlation coefficients for the comparison between plant phenolic compound production (anthocyanins and UV-absorbing compounds) in each plant tissue and reproductive outputs of *S. littorea*.** | | | | | | | | |
| --- | --- | --- | --- | --- | --- | --- | --- | --- |
| **Reproductive output** | **Petals** | | **Calyces** | | **Leaves** | | **Stems** | |
|  | **Ant.** | **UV-Abs.** | **Ant.** | **UV-Abs.** | **Ant.^1^** | **UV-Abs.** | **Ant** | **UV-Abs.** |
| Total flowers per plant | -0.1958 | 0.0651 | -0.0777 | -0.1461 | - | -0.0501 | -0.0615 | -0.1734 |
| Fruit set | 0.2523 | -0.0424 | 0.1637 | 0.1545 | - | 0.0665 | 0.1715 | 0.1261 |
| Ovules per flower | 0.0967 | 0.3726 | -0.2950 | -0.2715 | - | 0.2829 | -0.1474 | -0.1322 |
| Seed set | 0.1509 | -0.0753 | 0.1427 | -0.1413 | - | 0.0318 | 0.0309 | 0.0480 |
| Seed production per plant | -0.1493 | -0.0488 | 0.0313 | -0.1820 | - | 0.0180 | 0.2122 | 0.1791 |
| Pollen per anther | -0.2642 | 0.0035 | -0.5472 | -0.5542 | - | 0.1814 | -0.3960 | -0.2004 |
| ^1^ Anthocyanin concentrations in leaves were not considered in correlation analyses because of their almost complete absence in leaves. | | | | | | | | |
