# Supplementary figures and images for: UV radiation increases phenolic compound protection but decreases reproduction in Silene littorea
Source: PLoS One. 2020 Jun 18;15(6):e0231611. doi: 10.1371/journal.pone.0231611 (PMC7302690; doi:10.1371/journal.pone.0231611)

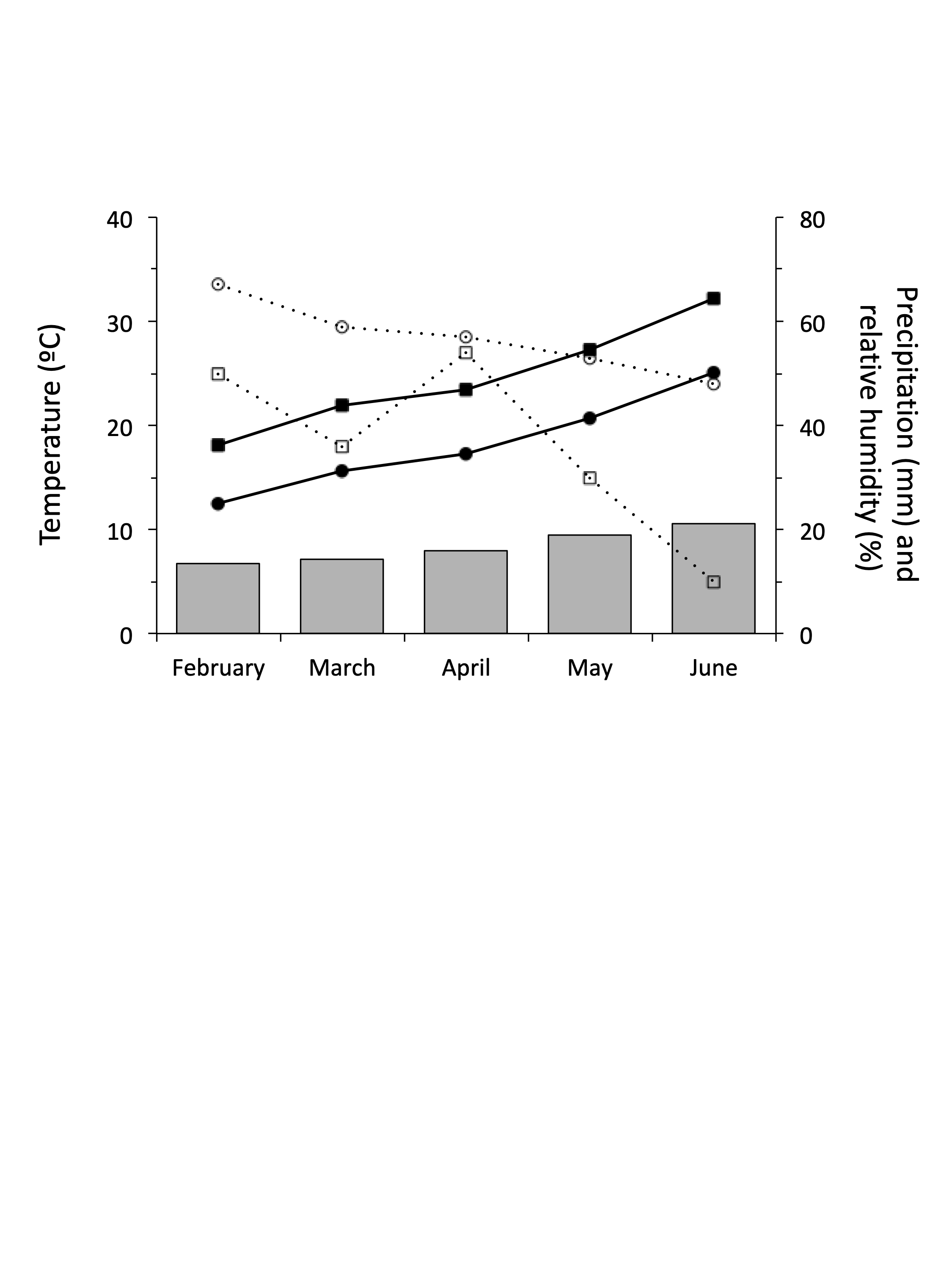

Supplement: S1 Fig — Filled circles, average temperature (°C); filled squares, maximum temperature (°C); empty circles and dashed line, relative humidity (%); empty squares and dashed line, precipitation (mm); bars, daytime duration (hours/day). Data from AEMET (State Meteorological Agency from Spain) database. (TIFF) [file pone.0231611.s004.tiff]

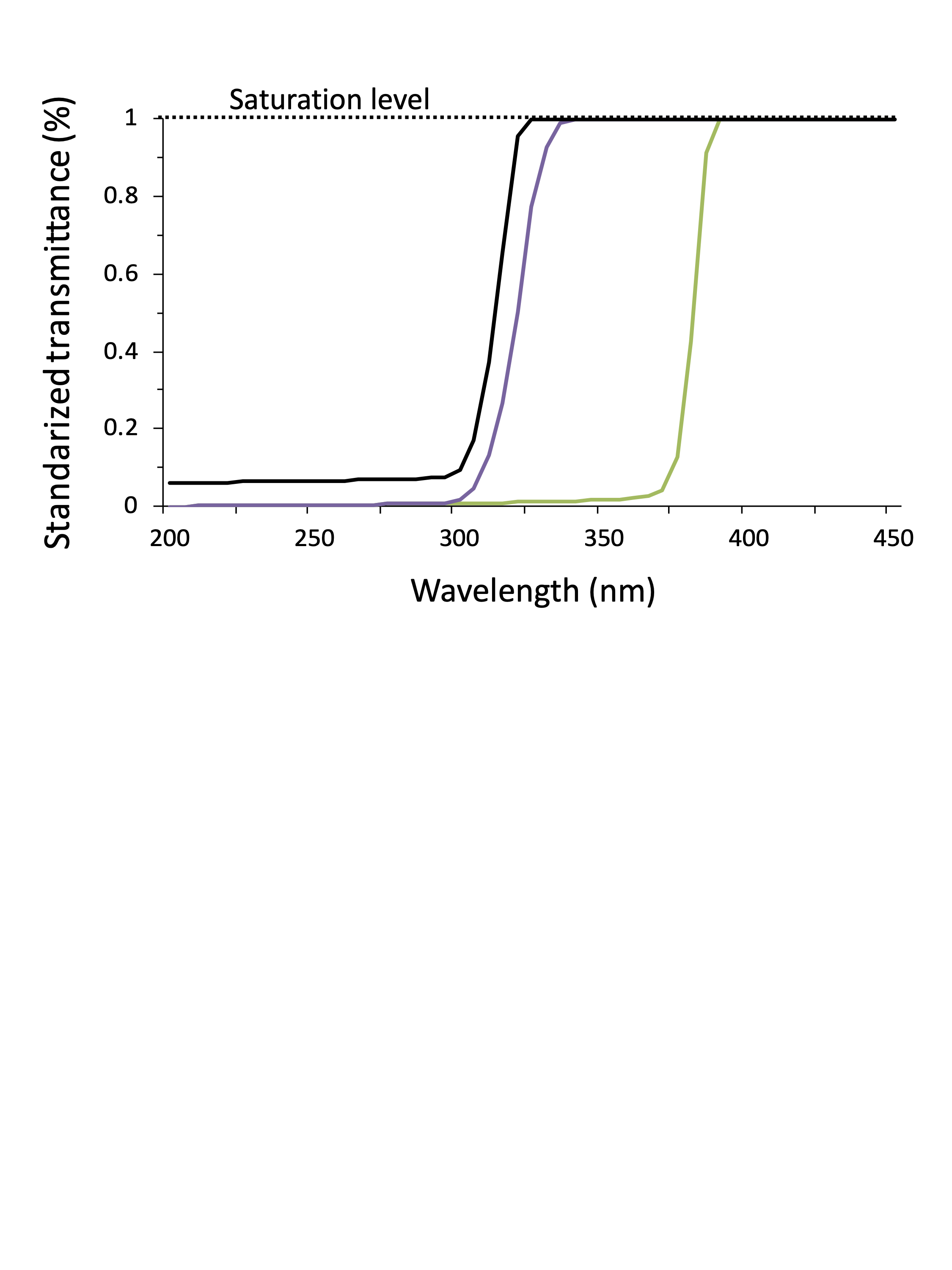

Supplement: S2 Fig — Methacrylate filter used in the UV-present treatment (purple line) allowed for the transmittance of significant portion of the UV irradiance, especially over the 335nm. Polycarbonate filter used in the UV-present treatment (green line) blocked the transmittance of UV wavelengths until the 385 nm, approximately. Natural sunlight is represented with a black line. Transmittance were measured using the portable spectrophotometer described in [63]. (TIFF) [file pone.0231611.s005.tiff]

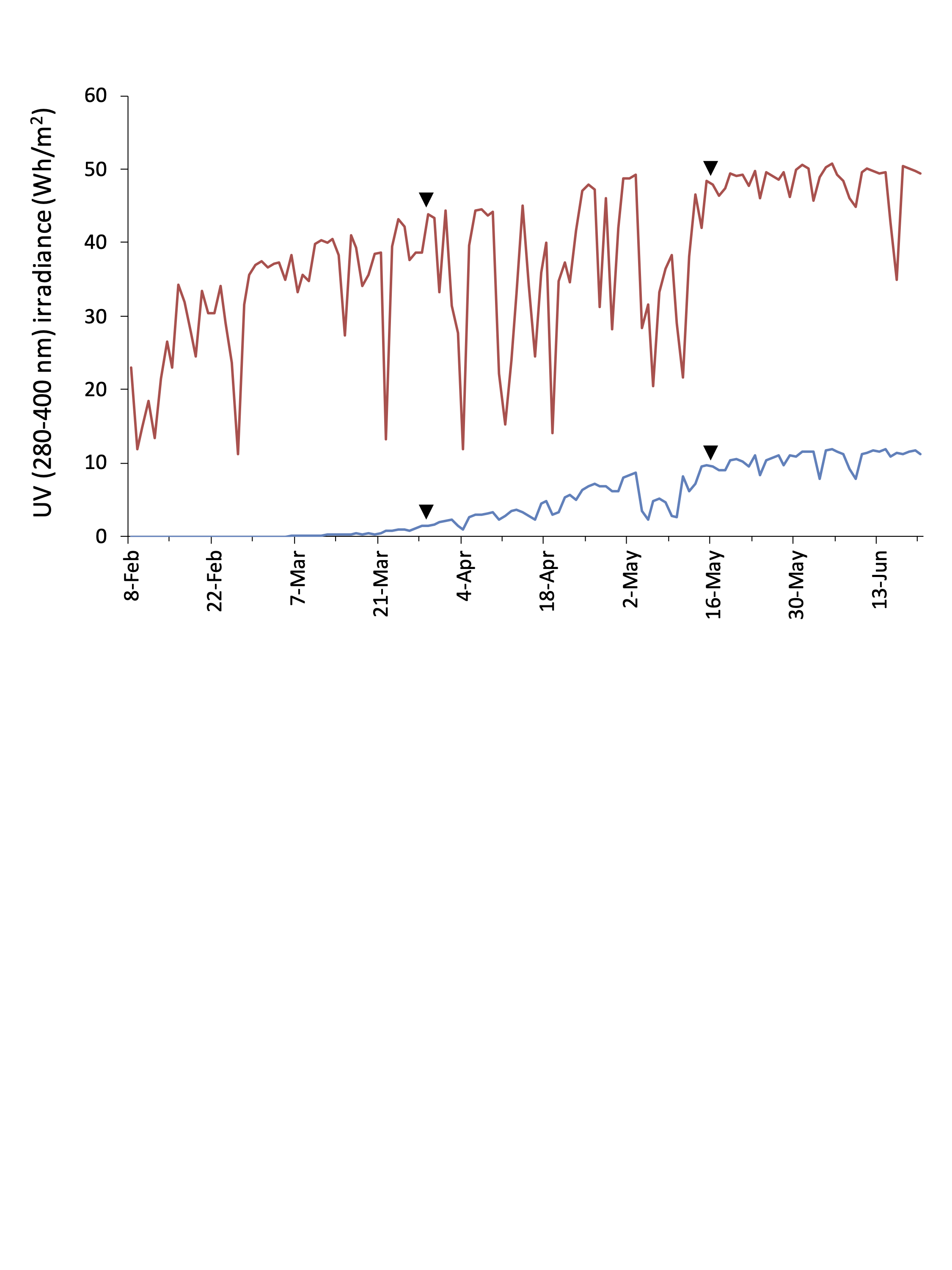

Supplement: S3 Fig — Blue and red lines represent UV radiation in predawn (7 AM) and in the afternoon (3 PM), respectively; triangles represent days when we performed the measurements of the photochemical efficiency of PSII (Fv/Fm) in the early flowering (March) and peak flowering (May). Data from HelioClim-3 database was provided by SoDa service. (TIFF) [file pone.0231611.s006.tiff]
